# Internet-delivered psychological treatment of functional gastrointestinal disorders in youth: Study protocol for exploration of cognitive biases

**DOI:** 10.1192/j.eurpsy.2022.1063

**Published:** 2022-09-01

**Authors:** E. Nielsen, K. Kallesøe, E. Bjerre-Nielsen, T. Gehrt, M. Bonnert, L. Frostholm, C. Rask

**Affiliations:** 1 Aarhus University, Department Of Clinical Medicine, Aarhus N, Denmark; 2 Aarhus University Hospital, Department Of Child And Adolescent Psychiatry, Psychiatry, Aarhus N, Denmark; 3 Aarhus University, Department Of Psychology And Behavioural Sciences, Aarhus C, Denmark; 4 Karolinska Institute, Department Of Clinical Neuroscience, Stockholm, Sweden; 5 Karolinska Institute, Department Of Medical Epidemiology And Biostatistics, Stockholm, Sweden; 6 Aarhus University Hospital, Department Of Functional Disorders, Aarhus N, Denmark

**Keywords:** CBT, functional gastrointestinal disorders, Child and adolescents psychiatry, Cognitive Bias

## Abstract

**Introduction:**

Functional gastrointestinal disorders (FGID) are common in children and adolescents (CA), cause functional disability and high health care use. Internet based cognitive behavioral therapy (i-CBT) have shown promising effect in Sweden. The treatment is exposure based and target avoidance behavior. Cognitive biases regarding bodily symptoms are suggested to be part of development and maintenance of functional disorders in adults, and could therefore be an important treatment target. Little is known about cognitive biases in CA with FGID, and hence the potential importance, it is crucial to explore more in depth. This study is embedded in *The Danish FGID Treatment Study* which aims to test Swedish i-CBT treatment in a Danish context.

**Objectives:**

1) Examine cognitive biases in CA with FGID, compared with healthy controls. 2) Asses these biases before and after treatment for FGID to investigate changes and impact on treatment effect.

**Methods:**

We developed a novel experimental approach to examine possible cognitive biases in CA. It consists of a picture task and a FGID-specific version of the Health Norms Sorting Task. We will examine 60 CA with FGID, who are included in The Danish FGID Treatment Study before and after treatment. In addition we will perform the test on 100 healthy controls.

**Results:**

The results are expected to improve our understanding of maintaining cognitive factors in CA with FGID, and how these may affect outcome and change during treatment, and how they differ from the general population.

**Conclusions:**

This study can provide new targets for treatment approaches.

**Disclosure:**

No significant relationships.

